# Erratum: Cheng, Y.-C., et al. Evidence Supports PA Prescription for Parkinson’s Disease: Motor Symptoms and Non-Motor Features: A Scoping Review. *Int. J. Environ. Res. Public Health* 2020, *17*, 2894

**DOI:** 10.3390/ijerph17145024

**Published:** 2020-07-13

**Authors:** Yi-Chen Cheng, Chun-Hsien Su

**Affiliations:** 1 Department of Exercise and Health Promotion, College of Education, Chinese Culture University, Taipei City 11114, Taiwan; cyz29@ulive.pccu.edu.tw; 2 Graduate Institute of Sport Coaching Science, College of Education, Chinese Culture University, Taipei City 11114, Taiwan

The authors wish to make the following corrections to their paper published in IJERPH [1]: authors’ affiliation.

[Old author’s name and affiliation]:

^1^  Department of Exercise and Health Promotion, Colleage of Education, Chinese Culture University, Taipei City 11114, Taiwan; cyz29@ulive.pccu.edu.tw
^2^  Graduate Institude of Sport Coaching Science, Colleage of Education, Chinese Culture University, Taipei City 11114, Taiwan

* Correspondence: chsu@faculty.pccu.edu.tw; Tel.: +886-975-159-678

to the correct version, as follows:

[New Author’s name and affiliations]:

^1^  Department of Exercise and Health Promotion, College of Education, Chinese Culture University, Taipei City 11114, Taiwan; cyz29@ulive.pccu.edu.tw
^2^  Graduate Institute of Sport Coaching Science, College of Education, Chinese Culture University, Taipei City 11114, Taiwan

* Correspondence: chsu@faculty.pccu.edu.tw; Tel.: +886-975-159-678

The authors would like to apologize for any inconvenience caused to the readers by these changes.
